# Unraveling the Link: A Comprehensive Literature Review of Type 2 Diabetes and Menopause Onset

**DOI:** 10.7759/cureus.50743

**Published:** 2023-12-18

**Authors:** Anuja A Mohile, Radhika P Hedaoo, Sammita J Jadhav, Archana S Ainapure, Mansi V Patil, Nalini R Khatwani

**Affiliations:** 1 Nutrition and Dietetics, Symbiosis International (Deemed) University, Pune, IND; 2 School of Beauty, Wellness, Nutrition and Dietetics, Symbiosis Skills and Professional University, Pune, IND; 3 Nutrition and Dietetics, Symbiosis Institute of Health Sciences, Symbiosis International (Deemed) University, Pune, IND; 4 Pathology, Symbiosis Institute of Health Sciences, Symbiosis International (Deemed) University, Pune, IND; 5 Nutrition, Asha Kiran Hospital, Pune, IND

**Keywords:** age of onset of menopause, obesity, ovarian aging, diabetes, pre-menopause, menopause, t2dm

## Abstract

Women with diabetes mellitus (DM), a metabolic endocrine illness, may experience a variety of reproductive problems. The age at menopause onset has been extensively studied as a major predictor of women's health in the future; however, its relationship to diabetes in Indian women has received less attention. This literature review looked at the consequences of diabetes in women as well as the association between diabetes and the age at which menopause begins. The average age at menopause onset among women with type 2 diabetes mellitus (T2DM) has decreased globally. According to one Indian study, the average menopause age dropped to 45 years for 26% of women with T2DM. In the current review, 10 studies indicated that women with T2DM displayed an imbalanced hormonal profile resulting in an extended anovulatory period. Two investigations highlighted the significance of altered body composition of women with T2DM, thereby suggesting obesity as the primary risk factor of ovarian aging and early climacteric symptoms. T2DM may lower the average age at menopause onset; however, further research on Indian women is necessary. There is a need of studies on T2DM in premenopausal women are needed to demonstrate how the changes in body composition impact the age at which menopause begins. Delaying the onset of menopause in women with T2DM necessitates diet and lifestyle interventions to minimize ovarian aging and hormonal imbalance.

## Introduction and background

Age at the onset of menopause is a key variable that impacts women's long-term health outcomes. Despite the fact that the basic mechanisms triggering the beginning of menopause are still poorly understood, the age at which menopause occurs is influenced by a complex combination of many factors. Diabetes and its impact on age at the onset of menopause has not been examined a lot. Type 2 diabetes mellitus (T2DM) is one of the largest global health concerns that largely impacts metabolism and, thereby, affects the quality of life of individuals [1]. The reason that can be cited for this unprecedented growth and that too in a developing country like India is the Asia paradox, where there are a number of developmental changes in terms of increasing urbanization and economic progress, which also lead to changed standards of living and, more importantly, an overall nutritional transition [2]. Various Indian studies have been conducted to understand the incidence of T2DM in different age groups as well as between the genders. Although research on the incidence of T2DM in both genders has consistently demonstrated that men are more likely than females to develop the disease, the fact that the overall incidence rate of diabetes has doubled also suggests that T2DM is becoming more common in women [3]. Clinically significant sex differences are becoming more evident, and T2DM and its associated comorbidities are rising significantly. A worldwide review of gender differences observed in terms of complications and pathophysiology of diabetes indicated that men showed T2DM at a relatively younger age than women and with lower body mass indices compared to women [4]. Insulin resistance, occurring due to factors, such as hormonal imbalance, obesity, family history, modernization, reduced physical activity, and faulty dietary habits, has been attributed to the increasing incidence of T2DM in women. Furthermore, it has been reported that although diabetes is not sex-specific, its impact on the female population is severe. Owing to reproductive considerations throughout a woman's lifespan, she experiences more pronounced changes in her body and hormones [5]. A woman undergoes several transitional hormonal changes throughout her reproductive span during menarche, pregnancy, lactation, middle age, and pre- and post-menopause. Previous studies in the last 10 years have shown that the reproductive life span of women affected with insulin-dependent diabetes mellitus is shorter, and the process of ovarian aging may lead to early climacteric symptoms. Menarcheal disturbances, oligomenorrhea, and amenorrhea leading to fertility disorders were observed in 32% of women in a study in which these women were diagnosed with T1DM [6]. The focus of the impact of T2DM has been more on the nephrological, neurological, and cardiovascular function and the reproductive function is comparatively less explored. A European Prospective Investigation into Cancer and Nutrition (EPIC) study of five countries depicted that the prevalence of diabetes in 119613 women was as high as 75% [7]. Thus, given the increasing number of women with diabetes, it is crucial to identify the impact of T2DM on their reproductive system. Existing T2DM and its correlation with menstrual disturbances, irregularities, the impact of insulin resistance on reproductive function, and the age of onset of menopause have not been evaluated to a great extent. Recent years have also seen an increase in the importance of pre-existing T2DM and its relationship with menstrual irregularities, disruptions, and the influence of insulin resistance on reproductive function. According to evidence on women with T2DM, ovarian aging and premature menopause may generally cause lengthy anovulatory episodes [6]. The longer a woman has diabetes, the more critical it is to understand the particular reproductive paths taken by her. Identifying patterns of reproductive aging may help identify individuals who are most at risk for chronic illnesses, as women with diabetes are more likely to have these problems [6]. There are ample studies that have explored the incidence of T2DM and other complications post-menopause. However, there is a dearth of studies which have explored T2DM leading to declining age of onset of menopause. The primary objective of the current review is to ascertain an association between prevailing T2DM in women and the declining age at the onset of menopause. Thus, to comprehend the causes of the declining age at menopause, risk factors such as obesity, age at menarche, gestational diabetes mellitus (GDM), insulin resistance, PCOS and the role of estrogen will be explored in this review.

Methodology

**Figure 1:**
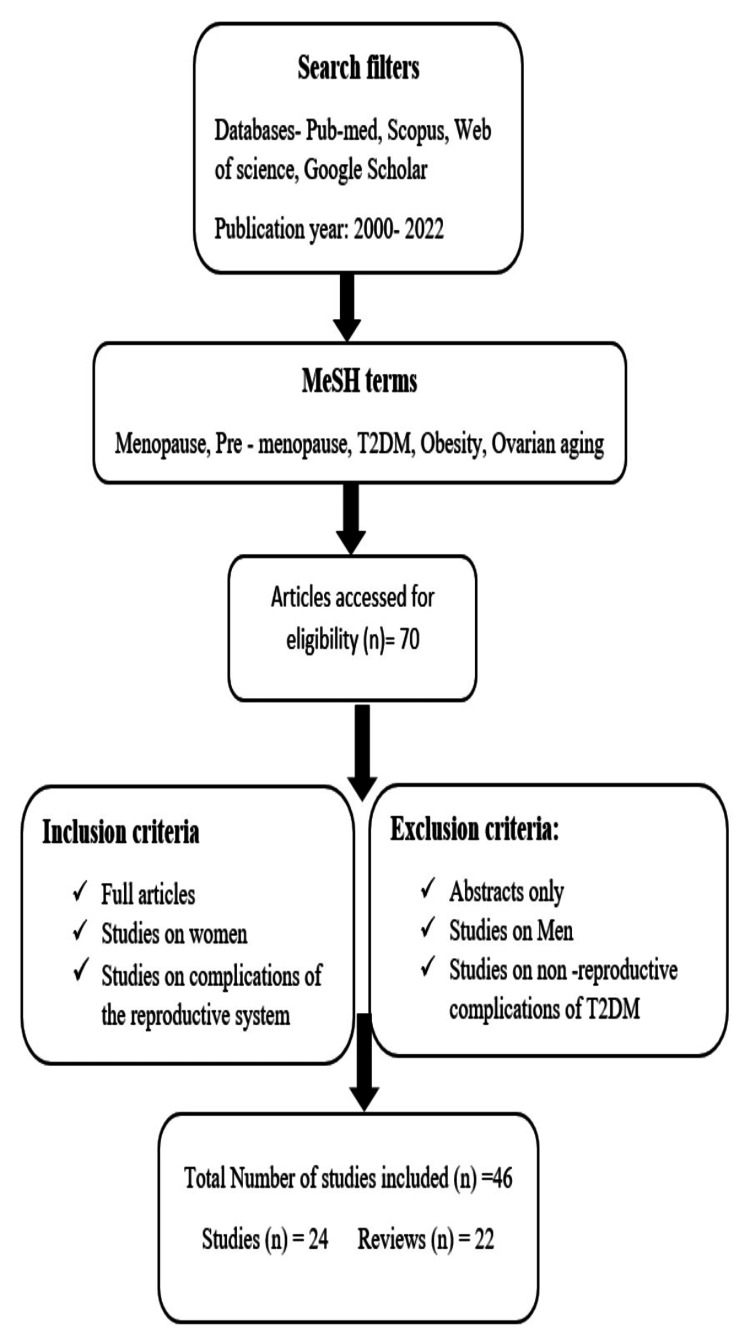
Methodology flowchart for search strategy T2DM: type 2 diabetes mellitus

Selected electronic databases, including Scopus, PubMed, Medline, Web of Science, and Google Scholar, were searched for scientifically published literature written between 2000 and 2022 to conceptualize the review. MeSH terms, such as, T2DM, age at onset of menopause, ovarian aging, obesity and pre-menopause were combined and applied. Studies were screened and investigated, with the objective of demonstrating that the prevalence of T2DM induced a decline in the age at onset of menopause. The studies that represented complications other than those associated with the reproductive system were excluded. Eight systematic reviews, 24 longitudinal, observational studies, and cross-sectional studies examined the relationship between obesity, early menarche, GDM, and polycystic ovary syndrome (PCOS) and the development of T2DM. Ten studies discussed the accelerated process of ovarian aging due to insulin resistance. Two studies - one of which was conducted in India - deduced that among women with T2DM, the age at which menopause begins to fall. Three systematic reviews evaluated the relationship between age at onset of natural menopause and existing T2DM. The flow diagram given below (Figure 1) demonstrates the search strategy for the review.

## Review

1. Pathogenesis of early menopause and its association with T2DM

**Figure 2:**
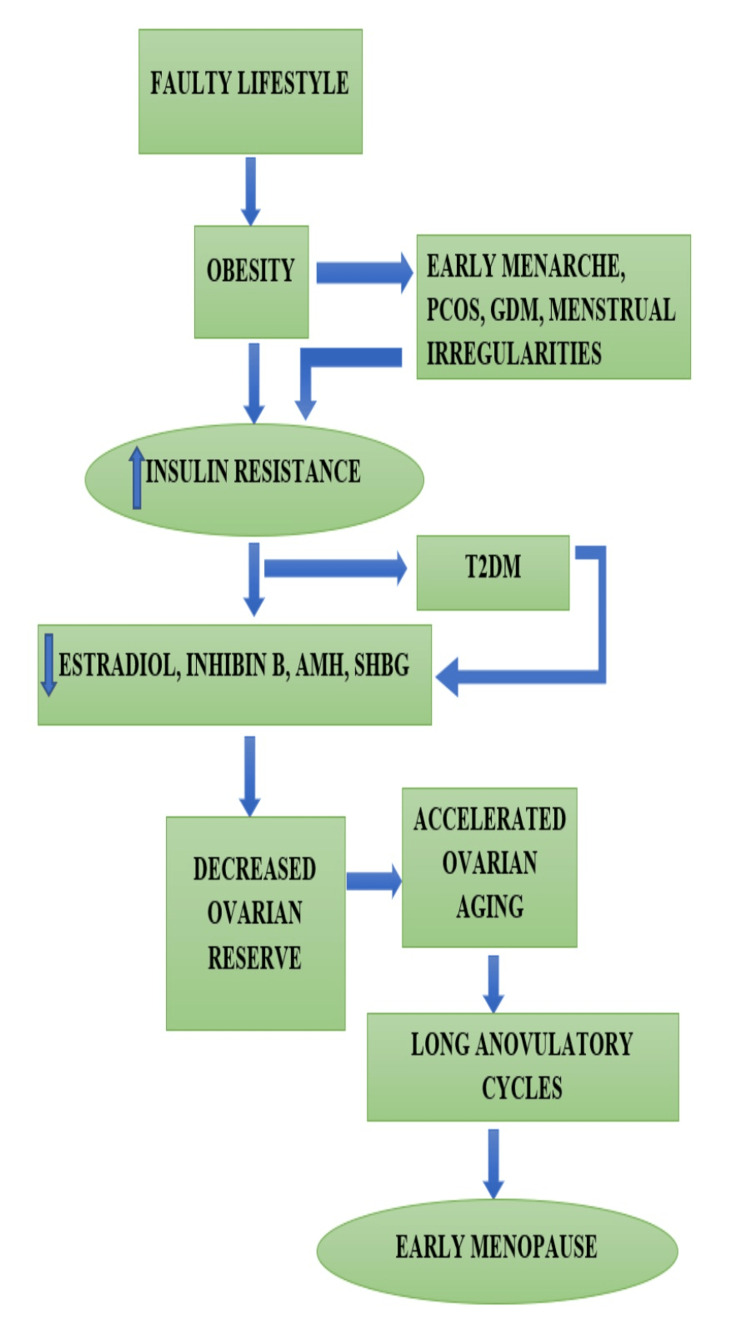
Lifestyle-induced T2DM and its correlation with premature onset of menopause Image Credit: Ms. Anuja Mohile T2DM: Type 2 diabetes mellitus; PCOS: Polycystic ovarian syndrome; GDM: Gestational diabetes mellitus; AMH: Anti-Müllerian hormone; SHBG: Sex hormone binding globulin

Menopause is an inevitable physiological change in every woman’s reproductive lifespan, but its onset timing may differ significantly. Globally, the age of onset of menopause usually ranges from 40 to 60 years, with 51 years being the average age. The age of onset is determined by natural influences, such as genetic and environmental factors. Similarly, the chronicity of metabolic disorders may also have an impact on the age of onset of menopause. The risk of menstrual irregularities, imbalances, and early menopause intensifies with a deranged metabolic profile. Due to unhealthy lifestyle choices that result in obesity and elevated blood glucose levels, there is a growing incidence of T2DM among the young reproductive female population. The imbalance of the blood glucose parameters directly impacts the hypothalamic-pituitary-ovarian axis, and may initiate insulin resistance contributing to a number of menstrual irregularities as early menarche and polycystic ovarian syndrome in young adolescent girls. The risk of developing T2DM in women with GDM was 33% high in an Indian study conducted in Bangalore on 128 women [5]. The resultant insulin resistance leads to decreased estradiol production, decreased production of sex hormone-binding globulins, and loss of ovarian reserves, leading to long anovulatory cycles and eventually may decline the age at onset of menopause [7] Inhibin B and anti-müllerian hormone (AMH) are considered to be significant markers of reproductive aging where inhibin B levels indicate the ovarian activity and the AMH levels are used to determine the ovarian reserves [8]. A longitudinal analysis conducted on 3293 women with T2DM showed that women who developed T2DM displayed poor AMH status thus contributing to the T2DM causing ovarian aging [9]. In an investigation including 162 healthy and T2DM-affected women, ovarian volume was shown to significantly decline along with levels of AMH and inhibin B, particularly in the T2DM-affected samples and the above 40 age group [10]. A recent study observed a lowering of estradiol levels in T2DM women before 40 years of age as compared to healthy controls where the estradiol levels showed a drop after the age of 45 years substantiating expedited ovarian aging thereby suggesting an early menopause commencement [11]. Consequently, the aforementioned research contributed to the formulation of a pathophysiology diagram for the onset of early menopausal symptoms because of the hormonal imbalance brought on by T2DM and lifestyle choices together (Figure 2).

2. Risk factors associated with T2DM and a decline in the age at onset of menopause

The incidence of T2DM in women is on the rise, which indicates that future metabolic complications will also increase if timely intervention strategies are not applied from nutritional and medical points of view. This section deals with the possible causative factors of T2DM and their impact on the age of onset of menopause.

2.1 Obesity, T2DM and Its Impact on the Age at Onset of Menopause

Obesity, at any age, is considered a complex disease. It has a deteriorating effect on the body in a psychological, social, and physiological manner. The prevalence of obesity during the growth phase of childhood and adolescence is directly correlated with obesity in adulthood. The neuroendocrine impact of obesity in females includes early onset of puberty and menarche, as well as high rates of dysmenorrhea, premenstrual disorders, hyperandrogenism, and insulin resistance in the future [12]. A recent trend showed increased obesity along with early onset T2DM in the young adult population. Obesity has long been recognized as a predisposing factor for increased inflammation, dyslipidemia, and insulin resistance in individuals, which may further lead to the development of T2DM and other cardiovascular diseases. Initially, as obesity progresses, insulin resistance occurs as a result of local inflammation of adipose tissue. This insulin insensitivity leads to the uncontrolled release of non-esterified fatty acids, secretion of inflammatory cytokines, and imbalanced production of adipokines, resulting in lipoprotein metabolism and overall systemic insulin resistance [13]. Higher BMI in early life or childhood obesity could be predictive factors for T2DM in later life. Past data from 2006 show an increased prevalence of overweight and obesity in children in urban India, with a higher incidence of obesity in private schools than in government-funded schools [14]. A recent study showed that the rate of obesity was high in American Indian children, and it was observed that these children were exposed to diabetes at the intrauterine stage. Furthermore, due to the characteristic obesity features in the Asian population such as low muscle mass, high body fat percentage and adiposity, the risk of metabolic syndrome and insulin resistance at a normal BMI was proven to be high. In Asian girls, complications such as PCOS and insulin resistance associated with obesity were higher than in the Caucasian population. Physical inactivity was also considered to be a contributing factor in the development of obesity and its impact on reproductive health [15]. Thus, the current incidence of increased obesity rates, which creates insulin resistance in the young female population, may impact the future reproductive health and even the age at the onset of menopause in future women. Lee et al. conducted a study exploring the association between obesity and the risk of diabetes in pre- and post-menopausal women. It was observed that the risk of T2DM was higher in obese women in their pre-menopausal stage due to the inhibitory impact of ovarian aging. This led to poor estradiol production in addition to the already existing insulin resistance as a result of obesity. Non-obese premenopausal women maintained higher levels of estradiol than obese women. Although this study could not successfully interlink obesity, diabetes, and age at the onset of menopause, it showed a positive association between increased BMI and the risk of developing T2DM in women and its impact on ovarian aging [16]. Nevertheless, more research is required to determine whether obesity and T2DM together cause early menopause.

2.2 Menarcheal Age, Duration, Length of the Cycle and Its Association With Obesity, T2DM and Early Menopause

Menarche is a critical point in a woman’s life, as it is considered a marker of her capability to reproduce and is different from puberty, which is marked by the development of secondary sexual characteristics. The age at menarche is determined by a number of factors such as body fat percentage, race, nutrition and physical activity status, BMI, and mother’s age at menarche [17]. Frisch and Revelle proposed the hypothesis of critical weight for menarche in 1969, in which it is assumed that there is a critical body weight at which only the period of menarche is marked. A few observations made as early as 1969 show that early menarche is seen frequently in obese young girls, as they tend to achieve the critical weight criteria at an early age. Factors, such as higher subcutaneous fat levels and increased BMI at the prepubertal age of 5-9 years, also influence the early occurrence of menarche [18]. Through their review of childhood obesity, researchers have shown that particularly in industrialized nations, childhood and adolescent obesity rates are rising rapidly to epidemic levels. The observation that overweight female children typically mature earlier than lean children has led to the theory that the level of body fat may initiate neuroendocrine processes that result in the onset of puberty. These children also showed higher adrenal androgen levels which were involved in the enhanced growth [19]. Steinberger et al. observed in their cross-sectional study of 342 children among 11-14 years of age. It was observed that girls had higher leptin levels than boys during puberty and this corresponded with alterations in the body fat percentage too. It was also observed that young girls with elevated leptin levels progressed faster, and presented an earlier onset of puberty. A constant elevated leptin was positively linked to insulin sensitivity and hyperinsulinemia in young children indicating the increased risk of metabolic complications in the adult stages [20]. A cohort prospective study of a sample size of 27,779 individuals from eight different European countries showed that women with T2DM showed a history of early menarche and elevated BMI. Furthermore, the findings of the study suggested that early puberty has an impact on metabolism in a partial manner, and there are a few other biological pathways apart from adiposity alone [21]. Xiao et al., observed through their meta-analysis that the age of menarche is declining and this concurs with the incline of obesity throughout adolescence as well as adulthood [22]. In one specific investigation, women between the ages of 20 and 30 who had a BMI between 18 and 29 kg/m^2^ were categorized into groups according to their age at menarche. Women in the early menarche category displayed distinguishing features such as higher BMI, lowered insulin sensitivity, and increased abdominal and truncal adiposity. Further, the study also indicated an association between the early age of menarche and increased fat levels among young women in their adult stage [23]. Thus, the risk of increased insulin resistance due to consistently high levels of body fat predisposes individuals to T2DM. A prospective cohort study was conducted in which pre-menopausal women self-reported their age at menarche. Other body parameters, such as BMI, waist circumference, total body truncal fat, fasting glucose, insulin, C-reactive protein, and sex hormone binding globulin (SHBG) levels, were evaluated. Women, classified as the early menarche category, showed elevated levels of fasting insulin, insulin resistance, and central fat accumulation during their adult life. A positive association between early menarche and the development of T2DM could be developed, but the correlation of early menarche leading to T2DM, which may further predispose a woman to early menopause, could not be confirmed [24]. The impact of early menarche on the onset of menopause has been extensively studied and a multinational study pooled data collected from populations of Japan, Scandinavia and Australia. More than 20 studies conducted on more than 50000 women have concluded that early menarche is a strong risk factor for the occurrence of premature menopause and the associated metabolic risks [25]. The length of the menstrual cycle also indicates the risk of T2DM. A prospective cohort study of 101073 female nurses aged 18-22 years of age indicated that the risk of T2DM was increased in women whose menstrual cycles were irregular in terms of length. The average menstrual cycle in this study was 26-31 days, where 40 days and above were considered oligomenorrheic. After a follow-up period of eight years, 507 patients were confirmed to have T2DM. In other cases, the relative risk for the women who reported their menstrual cycle length between 26 and 31 days was not elevated compared to the women who reported a shorter menstrual cycle of less than 21-25 days or slightly longer cycles of 32-39 days. However, the highest relative risk of T2DM was associated with women who reported long menstrual cycles of 40 or more [26].

2.3 Gestational Diabetes Mellitus (GDM) and Development of Early Menopause

GDM's detrimental effects on mother and child health have made it a growing public health concern in recent years. Short-term complications during pregnancy are exacerbated by GDM, whereas long-term complications during pregnancy include an enhanced risk of T2DM for women and a range of cardiometabolic illnesses for newborns [27]. The incidence of gestational diabetes has been associated with factors such as early puberty, obesity, pre-pregnancy nutritional status, and future risk of T2DM. A South Asian meta-analysis showed that the risk of developing T2DM was 10 times higher in women who were affected by GDM [28]. Persistent exposure to estrogen, in the case of early menarche and lowered SHBG in adulthood, plays a role in the development of GDM [21]. Findings of a meta-analysis developed by Xiao et al. suggested that early menarche had an important role in the development of GDM and it could be used as a marker to predict the occurrence of GDM. However, additional prospective studies were needed to confirm the same [22]. Further, a study conducted based on medical history of pregnancy such as GDM or hypertension and vasomotor symptoms in midlife could not very clearly bring out any positive association to prove the early onset of menopause in affected women [29]. Thus, more research is required to associate GDM with early menopause.

2.4 Polycystic Ovarian Syndrome (PCOS), T2DM, and Its Impact on Age at the Onset of Menopause

Among women of reproductive age, PCOS is one of the endocrine and metabolic illnesses that are most frequently reported. It is a diverse disorder marked by symptoms of ovarian dysfunction and androgen excess in the absence of another diagnosis. In India, using the Rotterdam and Androgen Excess Society criteria, the pooled prevalence of PCOS was determined close to 10% [30]. PCOS is linked to increased insulin resistance and, thereby, a higher probability of T2DM development. In a meta-analysis, it was found that the risk of developing T2DM in the presence of PCOS was three times higher in obese women belonging to Asian ethnicity and they displayed a distinctive morphology of ovaries that is similar to the polycystic ovaries [31]. In a study of North Indian women, with or without T2DM, 61% of them showed the presence of polycystic ovaries as compared to the controls. As per the study, these women showed PCOS along with insulin resistance and T2DM. Women with PCOS, even in the absence of T2DM, have a typical ovarian morphology which may accelerate the onset of T2DM [32]. Owing to PCOS, insulin resistance may lead to hyperinsulinemia and T2DM, which is also accompanied by obesity. Hypothetically, this interlinked route may ultimately impact reproductive functionality. However, this needs to be understood in depth through longitudinal studies to determine how PCOS in the presence of T2DM might have an impact on the decline in the age of onset of menopause [31].

2.5 Interplay of Insulin and Estrogen in T2DM Women

The symptoms of an existing metabolic disorder are worsened by menopause because it is marked by a decrease in estrogen production. As estrogen levels diminish in postmenopausal women, insulin resistance develops rapidly and becomes more severe [33]. Estrogen is a protective shield that women have, which declines with the onset of menopause. Independent of age, menopause may increase the possibility of developing insulin resistance because it lowers the level of circulating estrogen in the blood. Corresponding to these findings, a meta-analysis that compared the insulin resistance characteristics of pre- and post-menopausal women depicted that the post-menopausal women showed higher levels of insulin resistance [34]. This theory is supported by evidence that surgically induced menopause increases the risk of developing insulin resistance and other metabolic illnesses. Thus, the protective effect of estrogen against various metabolic diseases benefits healthy premenopausal women [35]. With the onset of menopause, metabolic abnormalities, such as insulin resistance, frequently become much more severe. This effect can be attenuated with hormone replacement therapy. A meta-analysis of the available data demonstrated that exogenous estrogen, when administered to women undergoing estrogen replacement treatment, significantly improves insulin sensitivity and lowered the risk of diabetes. De Paoli et al. have further evaluated the beneficial role of estrogen in association with insulin sensitivity. The glucose homeostasis of insulin-sensitive tissues is controlled by estrogen [36]. Although the link between insulin-sensitive women and the decline of age at the onset of menopause has not been established by the study, it can be theorized that women with T2DM have insulin resistance and therefore, may depict declined estrogen metabolism which might accelerate the arrival of menopause. A cross-sectional investigation of the impact of hormone replacement treatment on metabolic syndrome in Korean women with or without diabetes demonstrated that estrogen significantly reduced the risk of metabolic syndrome in both groups [37]. Insulin resistance, a principal characteristic of metabolic syndrome, was commonly observed in males as compared to premenopausal women of the same age and it was proposed that the risk of T2DM in healthy women was assuaged due to the protective effect of estrogen. Further, in the same review, the decline in insulin sensitivity, increased inflammatory markers and deranged lipid profile due to menopause or ovariectomy were depicted in rats [38]. Additionally, estrogen reduction can have a major impact on energy metabolism and the overall metabolic equilibrium. According to a Chinese study, T2DM incidence dropped by two years for every year when menopause was delayed [39]. All the aforementioned studies implicate the role of estrogen as well as the interplay of estrogen and insulin in women achieving menopause in a natural way at a normal age. An in-vivo study conducted in female insulin-resistant rats concluded that insulin levels play a role in maintaining menstrual cycle health throughout a woman’s life cycle. It was observed that insulin-resistant female rats maintained regular but short estrous cycles, indicating minor changes in the hypothalamic-pituitary-gonadal (HPG) axis, ultimately affecting the menstrual and reproductive cycles. Polyovular follicles, early progression of gestation, and complications in the later gestational phase were also observed due to the prevalence of insulin resistance. Although menopause or the impact on insulin resistance during menopause was not discussed in the study, it can be hypothesized that insulin signaling plays a very important role in the maintenance of the reproductive cycle, where menopause is the last but a crucial stage where the impact of insulin resistance needs to be researched deeply [40].

2.6 Ovarian Aging

Menopause is the climacteric stage in the process that is referred to as ovarian aging. The onset of cycle irregularity and the ultimate cessation of menstruation are determined by age-related decline in follicle counts. The gradual loss of fertility and eventual development of natural sterility are caused by a concurrent drop in oocyte quality. As the body ages, there is a progressive loss of physiological stability, and a number of degenerative changes accumulate, which hinders normal functionality, leading to heightened susceptibility to all infections and diseases. Ovarian aging includes all such changes taking place in the ovaries, which may be due to inevitable or pathological reasons. Pathological ovarian aging leads to premature ovarian insufficiency (POI), which can be classified into primary and secondary POI. Chromosomal abnormalities, enzyme shortages, and autoimmune diseases may contribute to the etiology of primary POI. An unhealthy lifestyle, reproductive surgery, surgical menopause, endocrine-disrupting substances, viral and infectious disorders, and other variables are linked to secondary POI [41]. The burden of T2DM is increasing owing to the substantially increased frequency of the condition worldwide, particularly in younger persons. Theoretically, T2DM results from a poor lifestyle and therefore may predispose a person to the secondary type of POI [42]. The term "ovarian aging" generally refers to the aging process of the female ovary, which is followed by a loss in ovarian follicle quantity and quality as well as granulosa and theca cell dysfunction. In a retrospective study conducted on 18 to 80-year-old female inpatients with or without T2DM, the estrogen, luteinizing hormone (LH), and follicle-stimulating hormone (FSH) levels of 964 T2DM patients and 263 controls were analyzed to conclude that the estrogen decline phase begins at the age of 40 in women with T2DM and was continuously low by the age of 45 years to show that ovarian aging was accelerated as compared to the controls thereby indicating that the aging-related changes in sex hormones of the patients accelerate ovarian aging. Chronic inflammation was suggested as the underlying mechanism contributing to T2DM's pathogenesis, which may lead to oxidative stress in the ovarian microenvironment. Reactive oxygen species build up in the ovaries as a result of increasing oxidative stress, which leads to a decline in oocyte quality and hastens ovarian aging [43]. Vascular damage, a typical T2DM consequence linked to aging, is another factor that along with dyslipidemia deteriorates and affects oocyte quality. The study has also discussed advanced glycation end products (AGEs) that are strongly linked to deteriorating ovarian function through the pro-inflammatory pathway, and T2DM is known to be associated with an increased rate of AGE accumulation [11]. The issue of accelerated ovarian aging and its negative effects on patients must be addressed because the number of female patients with T2DM being diagnosed at younger ages is sharply increasing. There is a paucity of research demonstrating a clear correlation between T2DM and menopause onset age. Factors such as childhood obesity, persistently high BMI, early menarche, GDM, and irregular length of menstrual cycle are all factors that increase the risk of T2DM; however, little data are available regarding these conditions and their direct impact on the age of onset of menopause in women with T2DM.

3. Association of T2DM age onset of menopause in T2DM women

Menopause is a complex phenomenon marked by the cessation of the monthly menstrual cycle due to loss of follicular function, which could also be described as ovarian aging. Post-menopause, irrespective of age, a number of metabolic derangements occur, leading to complex diseases, such as T2DM, cardiovascular diseases, cancers and metabolic syndrome [44]. The risk of future illness is significantly influenced by the timing of menopause. Women who experience menopause at a young age have a higher risk of developing cardiovascular disease and osteoporosis [45]. Given the current circumstances, women are experiencing an increase in the global incidence of T2DM due to a number of common etiological factors that have been previously highlighted. This increases the risk of a large percentage of the female population experiencing severe aging climacteric symptoms. Very few studies have investigated the average age of natural menopause in women with T2DM as the onset of T2DM is usually anticipated after menopause. Sasi Sekhar et al., through their study on metabolic characteristics of 600 postmenopausal non-diabetic (n=300) and diabetic (n=300) Indian origin women, concluded that the age of onset of menopause was early in a total of 212 women. Out of these 212 women 158 women were diagnosed with T2DM before their menopause itself whereas 54 women were non-diabetic before the onset of menopause. The specific metabolic characteristics of these 158 women were high BMI and an imbalanced metabolism of glucose and insulin. Furthermore, these women patients had a younger average age of onset of menopause than non-patients (44.65 versus 48.2 years; p = 0.01). In addition, early menopause (before age 45) was more prevalent in 50% of women with T2DM than in 17% of women without T2DM. These results are in line with those of a study of 5669 women in Latin America, which found that the prevalence of early menopause was more than three times greater in 40 to 44-year-old women with T2DM (n = 410) compared to women without (n = 5669). An age-segmented analysis of the data revealed, that in women between the ages of 40 and 44 years, the chance of going through menopause for women with diabetes was about three times higher than the risk for non-diabetic women, but this difference disappeared in older women. This information may enable us to predict that a subset of women with diabetes will experience early menopause because of the metabolic side effects of the condition [45].

In a systematic review, Yazdkhasti et al. have discussed the impact of T2DM on the reproductive system to be complicated and it affects the organs at a microvascular level. Further, the review observed that women with T2DM portray diverse patterns of reproductive complications with a common tendency of decline in the age of onset of menopause [46].

With the rising prevalence of diabetes in women at younger reproductive ages, longitudinal studies assessing putative causes of ovarian aging in women with diabetes are required.

Studies demonstrating an obvious association between T2DM and early menopause are uncommon. Hence, the basis for this analysis is to determine a correlation between the aging of the ovaries, obesity, early menarche, and other predisposing variables that raise insulin resistance, thereby declining the age at the onset of menopause.

Table 1 given below summarizes the literature on the risk factors for early menopause in women with T2DM.

**Table 1:** Summary of risk factors involved in T2DM that decline age at onset of menopause T2DM: Type 2 diabetes mellitus

Risk factors for type 2 diabetes mellitus and its association with early onset of menopause	Studies found	Results	Future prospects
Obesity leading to type 2 diabetes mellitus (T2DM)	[12-16]	Obesity is strongly associated with the development of insulin resistance, type 2 diabetes and further reproductive issues. The incidence of early menopause is not directly correlated with obesity-induced type 2 diabetes (T2DM).	To develop this link, more research is needed. Intervention studies can be carried out to lower the incidence rate of T2DM and obesity.
Early menarche and increased risk of T2DM	[17-26]	Elevated leptin levels in childhood obesity are linked to early menarche. An increased risk of metabolic diseases such as type 2 diabetes occurs when obesity persists throughout adulthood. One significant factor that contributes to T2DM risk may potentially be the cycle's length and duration. There is a positive correlation between early menarche and early menopause. While a positive correlation has been established between obesity and early menarche, which subsequently causes type 2 diabetes, there hasn't been much research done on the positive relationship between T2DM and the development of early menopause.	Research on early intervention to postpone menarche by nutritional management of obesity and type 2 diabetes should be carried out.
Gestational diabetes mellitus and increased risk of T2DM	[21,27-29]	For patients with GDM, the risk of developing type 2 diabetes is ten times higher. In a similar vein, early menarche may raise the chance of both T2DM and GDM. Women who have had GDM in the past may experience early vasomotor symptoms during menopause.	It is essential to do research to determine the relationship between early menopause and gestational diabetes mellitus. The focus should be on interventions aimed at reducing the increasing number of GDM cases, which will in turn lower the incidence of T2DM.
Polycystic ovarian syndrome and insulin resistance	[30-32]	Asian women are more likely to develop PCOs because of their unique ovarian shape, which also raises the risk of insulin resistance in them. Research demonstrating a connection between early menopause and PCOS is lacking.	It is important to conduct research on the possible link between PCOS and early menopause. Dietary strategies should be devised to counter PCOS and its aftereffects.
Interplay of insulin and estrogen	[33-40]	The protective hormone, estrogen levels drop with the onset of menopause, which has a significant effect on women's metabolic processes. Insulin resistance, a characteristic feature of T2DM and the metabolic syndrome may lower estrogen levels, which may worsen ovarian and thereby the reproductive health of women.	Early education regarding Type 2 Diabetes (T2DM) and its effects on later stages of life is important for women. Additional comprehensive research is needed to establish a correlation between age at menopause onset, insulin, and estrogen.
Role of T2DM in ovarian aging	[11,41-43]	Secondary ovarian insufficiency occurring due to lifestyle changes, lack of activity and T2DM causes chronic inflammation in the microenvironment of ovaries. This may hasten the ovarian aging process and thereby decline the age at onset of menopause	Studies to depict this association directly need to be conducted. Health and nutrition intervention for women should be initiated at an early age.

## Conclusions

T2DM's implications for aging and reproductive health are multifaceted phenomena. The current analysis suggests that women with T2DM show a significant decline in their age at the onset of menopause. The incidence of T2DM in women has increased and therefore the future health implications need to be addressed to prevent the reproductive system-based repercussions which are often overlooked. The reproductive dysfunctions may occur because of over-exposure of insulin to the ovaries, which is a characteristic feature of hyperinsulinemia. This over-exposure of insulin to ovaries accelerates the aging process of ovaries finally causing a decline in the age of onset of menopause. It is therefore crucial to explore the trends of increased T2DM in women, its link with risk factors such as early menarche, obesity, PCOS and GDM, and its association with early menopause and its complications. Untreated early ovarian sufficiency reduces health and life expectancy. So far, it has been evidenced that metabolic complications arise due to the decreased estrogen level, in the case of menopause. This review has attempted to put forward the fact that prevailing T2DM in women can hasten the process of aging. This may lead to a decline in the age at onset of menopause which itself increases the risk of all metabolic complications. Therefore, our study confirms the significance of early diabetes diagnosis and treatment for a woman's long-term health and quality of life.
